# Environmental Remediation of Toxic Organic Pollutants Using Visible-Light-Activated Cu/La/CeO_2_/GO Nanocomposites

**DOI:** 10.3390/ma14206143

**Published:** 2021-10-16

**Authors:** Dhanapal Vasu, Yongsheng Fu, Arjunan Karthi Keyan, Subramanian Sakthinathan, Te-Wei Chiu

**Affiliations:** 1 Department of Materials and Mineral Resources Engineering, National Taipei University of Technology, No. 1, Section 3, Chung-Hsiao East Road, Taipei 106, Taiwan; dvasukalyan@gmail.com (D.V.); karthikeyan100596@gmail.com (A.K.K.); 2 Key Laboratory for Soft Chemistry and Functional Materials, Nanjing University of Science and Technology, Nanjing 210094, China; fuyongsheng@njust.edu.cn

**Keywords:** visible light, degradation, wastewater, Rhodamine B, sunset yellow, cibacron red, nanocomposites

## Abstract

Environmental pollution is a major threat that increases day by day due to various activities. A wide variety of organic pollutants enter the environment due to petrochemical activities. Organic contamination can be unsafe, oncogenic, and lethal. Due to environmental issues worldwide, scientists and research communities are focusing their research efforts on this area. For the removal of toxic organic pollutants from the environment, photocatalysis-assisted degradation processes have gained more attention than other advanced oxidation processes (AOPs). In this manuscript, we report a novel photocatalysis of copper and lanthanum incorporating cerium oxide (CeO_2_) loaded on graphene oxide (Cu/La/CeO_2_/GO) nanocomposites successfully synthesized by hydrothermal techniques. XRD results showed the presence of dopant ions and a crystalline structure. FESEM images showed that the surface morphology of the synthesized nanocomposites formed a rod-like structure. The highlight of this study is the in-situ synthesis of the novel Cu/La/CeO_2_/GO nanocomposites, which manifest higher photodegradation of harmful organic dyes (Rhodamine B (RhB), Sunset Yellow (SY), and Cibacron Red (CR)). In Cu/La/CeO_2_/GO nanocomposites, the dopant materials restrict the rapid recombination of photoinduced electron–hole pairs and enhance the photocatalytic activity. The degradation percentages of RhB, SY, and CR dye solution are 80%, 60%, and 95%, respectively. In summary, the synthesized nanocomposites degrade toxic organic dyes with the help of visible light and are suitable for future industrial applications.

## 1. Introduction

Every living organism in the world depends on water. Moreover, only a small percentage of the freshwater in the world is available for drinking and agriculture [1]. However, the rapid growth of human activities, such as urbanization and industrialization, has had a major impact on water resources. Industries such as the textile, leather, paper, medical, pulp, and dyeing industries release their effluent into water resources without any purification, causing water pollution [1,2,3]. A higher amount of wastewater is generated by the textile industry than by other industries. Effluent from the textile industry contains a wide variety of dyes, which make the water colored, toxic, and carcinogenic for all living organisms. During the dyeing process, 1–20% of the dye is lost, and it is directly disposed of in water resources [4,5,6]. This wastewater contains high amounts of anthraquinone and azo and heteropoly aromatic dyes. Among the textile dyes, azo dyes are a special class of dye and are widely used, constituting 60–70% of all dye products [2,7,8,9,10]. Azo dyes are classified by their aromatic compounds, which include one or more azo groups (–N=N–). Therefore, the wastewater from that industry is highly harmful to human and aquatic life because the waste products have serious impacts on the environment [10,11,12].

To develop suitable techniques for the degradation and mineralization of dye-polluted wastewater, many techniques are applied to degrade dye effluent, as follows: (1) physical methods, such as membrane filtration, flotation, and sedimentation; (2) biological methods; and (3) chemical methods, such as chemical coagulation, photocatalysis, Fenton or Fenton-like oxidation, and ozone (O_3_) oxidation processes [13,14,15]. Although those treatment methods have some advantages, they cannot fully degrade organic contaminants in industrial wastewater [16]. Among the emerging techniques for textile effluent decontamination, advanced oxidation processes (AOPs) are capable of mineralizing non-biodegradable and toxic organic compounds [9,16,17]. AOPs are generally based on the in situ generation of more active reactive species (OH^•^, H_2_O_2_, O_3_, O_2_, and O_2_^•^^−^) [12,18]. In AOPs, the oxidation process is primarily responsible for the removal of textile effluents. In recent decades, metal oxide nanoparticles have attracted research efforts due to their high photocatalytic activity and stability. Particularly, nano-sized ceria (CeO_2_) photocatalysis for the oxidation of organic compounds has intrinsic advantages over other types of photocatalysis because of its properties [19]. Ceria is a ceramic material used in fields such as fuel cells, gas sensors, phosphors, polishing materials, energy storage devices, and catalysis.

For these applications, the research community constantly seeks to enhance the physicochemical properties of ceria to attain better performance. Ceria is an emerging photocatalyst for dye wastewater treatment, and it has a bandgap of 3.2–3.4 eV, which is similar to the bandgap of TiO_2_ [20,21]. Hence, ceria can be significantly enhanced by tuning the bandgap, size, interface, and surface structure via doping with trivalent and divalent cations in the crystal lattice. The reduction capability of ceria, from the Ce^4+^ ionic state to Ce^3+^ with the incorporation of oxygen vacancies, makes it a strong contender for use as a catalyst or photocatalyst. Copper oxide (CuO) is a narrow-bandgap (1.2 eV) p-type semiconductor material that shows excellent catalytic properties [22,23,24]. The incorporation of copper ions into the fluorite-type crystal structure of ceria can enhance its properties, such as the oxygen storage capacity, sintering temperature, and photocatalytic activity. A synergetic effect between Cu^+^/Cu^2+^ and Ce^3+^/Ce^4+^ results in higher interfacial redox activity, and at the same time, the catalytic ability of Cu-doped CeO_2_ is changed or enhanced [25]. Moreover, the important trivalent metal ions that are generally considered include Sm^3+^, Eu^3+^, Sc^3+^, Gd^3+^, and La^3+^, among others. La^3+^ has important applications in various fields, particularly in electronic devices, photocatalysis, and optical and solid oxide fuel cells. When trivalent La ions are doped into the ceria crystal lattice, the La^3+^ ions replace Ce^4+^ ions and create a charge imbalance, which introduces oxygen vacancies into the surface of the material. These surface defects are mainly responsible for the improvement of the photocatalytic properties of the photocatalyst. Moreover, an efficient strategy is to deposit the photocatalysts on conducting substrates to enhance the conductivity and dispersibility [26,27,28,29]. 

Graphene, as a two-dimensional, hexagonal, single-layered carbon, has attracted attention to non-metal catalysts. Anchoring Cu/La/CeO_2_ catalysts on graphene is an important solution for engineering the photocatalytic activity of Cu/La/CeO_2_. To the best of our knowledge, no Cu/La/CeO_2_/GO-related photocatalytic application has been achieved under visible light to date. In this study, we synthesized Cu/La/CeO_2_/GO nanocomposites through hydrothermal techniques. The photocatalytic ability of the synthesized nanocomposites was studied using three different organic dyes (RhB, SY, and CR) under irradiation with visible light. The role of reactive radicals was studied with the help of free radical scavenging techniques. A possible degradation reaction mechanism for RhB, SY, and CR dyes was explored to illustrate the reaction involved in photocatalysis. The results revealed that Cu/La/CeO_2_/GO nanocomposites have a high potential for degradation of organic compounds under visible-light irradiation.

## 2. Materials and Methods

Cerium (III) nitrate hexahydrate (Ce(NO_3_)_3.6_H_2_O), copper (II) nitrate trihydrate (CuH_6_N_2_O_9_), lanthanum (III) nitrate hexahydrate (La(NO_3_)_3.6_H_2_O), and sodium hydroxide (NaOH) were purchased from Sigma-Aldrich, Taiwan. Any other modifications and purifications were allowed for these materials. Moreover, double-distilled (DI) water was used in all experiments. 

### 2.1. Synthesis of Graphene Oxide (GO)

Modified Hummers’ preparation techniques were used to prepare the graphene oxide (GO) [30]. In this experiment, 10 g of graphite powder was introduced into concentrated sulfuric acid (100 mL) with 2 g of sodium nitrate and stirred in an ice-water bath to control the solution temperature. Then, 5 g of potassium permanganate was added to the above mixture, and the temperature of the solution was held at 20 °C until the mixture turned green. The mixed solution was transferred to another water bath with a temperature of 35 °C, and 400 mL of DI water and 20 mL of hydrogen peroxide solution were added to the solution before it was stirred for 1 h to extract potassium permanganate. Finally, the solution was washed and cleaned with DI water several times. The cleaned GO solution was dried and heated in a hot-air oven at 90 °C for 24 h. 

### 2.2. Synthesis of Cu/La/CeO_2_/GO Nanocomposites 

Hydrothermal techniques were used to synthesize Cu/La/CeO_2_/GO composites. Equal (0.1) molar ratios of copper, cerium, and lanthanum precursor were placed into separate beakers containing 20 mL of DI water. Those solutions were simultaneously stirred magnetically for 30 min to obtain homogenous mixtures. After that, the copper and lanthanum solutions were added dropwise into the cerium solution under magnetic stirring, and 0.2 M NaOH solution was added as a reducing agent. The mixed solution was steadily stirred for 1 h at ambient temperature. After stirring, 100 mg of reduced graphene oxide was added to the above solution, which was further stirred for 1 h The resulting composite solution was transferred to a statin-less, steel-lined autoclave and then heated in a hot-air oven at 120 °C for 12 h. After the hydrothermal process, the autoclave was cooled naturally at room temperature. The Cu/La/CeO_2_/GO composites were centrifuged and washed with DI water and ethanol several times, and then the obtained composites were dried in an oven at 90 °C for 24 h. The obtained powder composites were calcinated at 600 °C for 3 h. The crystalline structure, surface morphology, functional groups, and elemental surface chemistry of the synthesized composites were characterized by methods such as X-ray diffractometry (XRD) and field-emission scanning electron microscopy (FE–SEM). 

### 2.3. Characterization of Cu/La/CeO_2_/GO Nanocomposites 

The hydrothermally prepared nanocomposites were characterized with various techniques. The diffraction patterns of the nanocomposite were observed using X-ray diffraction (XRD, D2 Phaser, Bruker, CuKα radiation (λ = 1.54 Å)). The surface structure and topography were obtained with a field-emission scanning electron microscope equipped with an energy-dispersive X-ray spectroscope ((FESEM-EDX, JEOL, JSM-7610F, Tokyo, Japan), and (Hitachi Regulus 8100, JEOL JPS-9030, AlKα, Tokyo, Japan)), and by transmission electron microscopy (TEM, JEM-2100F, JEOL, Tokyo, Japan). In addition, the degradation of wastewater polluted with organic compounds was analyzed with a UV–visible spectrophotometer (SHIMADZU, UV-2600, Kyoto, Japan).

### 2.4. Photocatalytic Activity 

**Figure 1:**
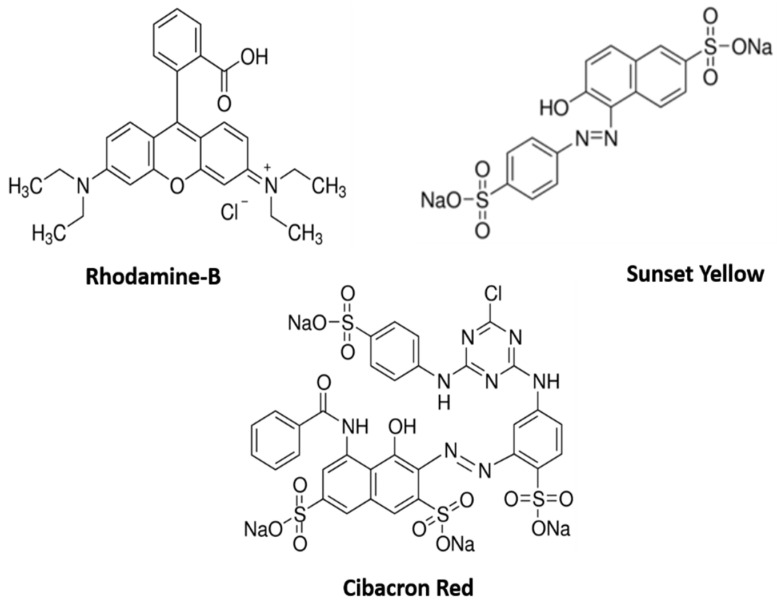
Chemical structure of three different dyes.

The photocatalytic performance of the hydrothermally synthesized nanocomposites in the degradation of three different organic dyes, namely, RhB, SY, and CY, (Figure 1) under visible-light irradiation was studied. The three different organic dyes were prepared with concentrations of 10^−4^ moles. A 50 W tungsten-halogen lamp (340–850 nm, UV to NIR) was used as the visible-light source. For the degradation process, 100 mL of dye solution was irradiated under visible light. For this experiment, 20 mg of nanocomposites was added to the dye solution, which was constantly stirred. Before the experiment, the dye solution with a catalyst added was stirred continuously at 30 min under the dark condition to acquire an adsorption–desorption equilibrium condition. The solution mixture was irradiated with visible light for 90 min under magnetic stirring, and every 10 min, 5 mL of the solution was extracted for the study of the degradation/decolorization of dye with a UV–visible spectrometer. Before the absorbance analysis, the extracted samples were filtered using Whatman filter paper to remove the photocatalyst.

## 3. Results and Discussion 

### 3.1. Study of the Crystalline Structure 

**Figure 2:**
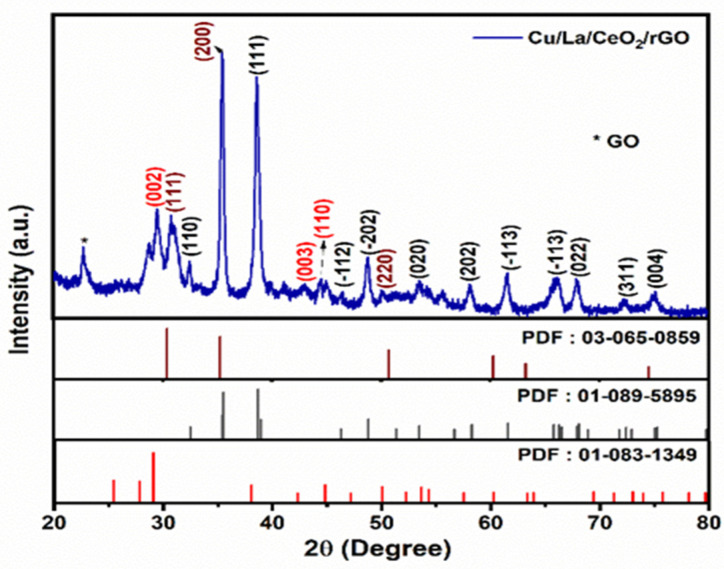
XRD analysis of Cu/La/CeO_2_/GO nanocomposites.

The XRD patterns of the hydrothermally synthesized Cu/La/CeO_2_/GO nanocomposites are depicted in Figure 2. The diffraction peaks of CuO were attributed to the (−110), (111), (−112), (−202), (020), (202), (−113), (022), (311), and (004) planes, which indicated the monoclinic structure of CuO crystals (JCPDS: 01-089-5895). The peaks due to CeO_2_ also indicated the (111), (200), and (220) planes, which were attributed to the cubic fluorite-structured CeO_2_ crystals (JCPDS: 03-065-0859) [19,20,21,22]. In addition, La_2_O_3_-related peaks were attributed to the (002), (011), and (003) planes, which were indexed to the hexagonal structure of La_2_O_3_ crystals (JCPDS: 01-083-1349) [23,24,25,26]. The GO-related peaks also presented 2θ at 22.74°. These results indicated that the dopant ions were successfully doped into the ceria lattice structure due to the differences between the ionic radii of the Cu^2+^ (77 pm), La^3+^ (103 pm), and Ce^4+^ (102 pm) ions, respectively. 

The nanocomposite crystalline size was also calculated using Scherrer’s equation:

D = kλ/(β_(1/2)_ cosθ) (1)

where the incident X-ray wavelength (1.54 Å) and crystalline factor (0.94) are denoted as λ and k, respectively, and the full width at half maximum (FWHM) of the peak and the Bragg angles are denoted as β and θ, respectively. The average crystalline size of the synthesized nanocomposites was approximately 16.43 Å. We concluded that the doping of Cu/La atoms decreased the crystalline size of the prepared nanocomposites based on the literature.

### 3.2. Surface Morphology and Topography Studies of Nanocomposites

**Figure 3:**
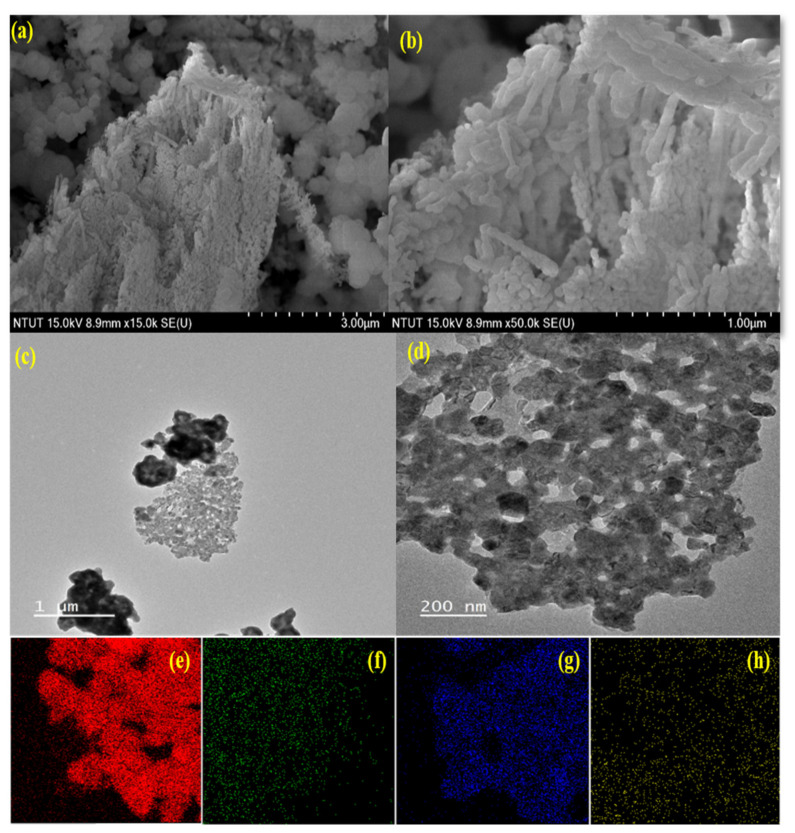
(**a**,**b**) Surface morphology of synthesized Cu/La/CeO_2_/GO nanocomposites. (**c**,**d**) TEM images for Cu/La/CeO_2_/GO nanocomposites, and elemental mapping for Cu/La/CeO_2_/GO nanocomposites: (**e**) Ce, (**f**) Cu, (**g**) La, and (**h**) C.

The Cu/La/CeO_2_/GO morphology and structures were studied by FE–SEM analysis (Figure 3a,b). The analysis revealed that the hydrothermal synthesis methods caused the nanocomposites of Cu/La/CeO_2_/GO to grow into rod-like structures of uniform size.

TEM images of Cu/La/CeO_2_/GO nanocomposites are shown in Figure 3c,d. Figure 3a,b presents TEM images of aggregated Cu/La/CeO_2_/GO nanocomposites showing that the particle size was small. In addition, the nanocomposites were irregular in shape, and their size was uneven. STEM–EDS elemental mapping showed the presence of all the doping materials, as shown in Figure 3e–h. It was concluded that the Cu, La, Ce, and C in Cu/La/CeO_2_/GO nanocomposites overlapped. 

### 3.3. Nanocomposites Surface Chemistry Studies

**Figure 4:**
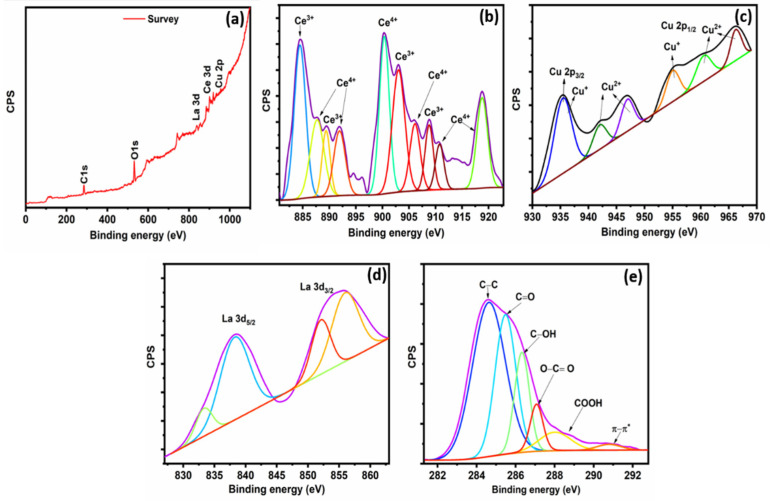
(**a**) The survey XPS spectrum; high-resolution XPS core-level spectra, (**b**) Ce 3d, (**c**) Cu 2p, (**d**) La 3d, and (**e**) C 1s spectra, respectively.

Figure 4 depicts the XPS survey and core-level high-resolution spectra of the Cu/La/CeO_2_/GO nanocomposites. XPS is highly surface sensitive, which allows identification of unknown materials and oxidation states on the surfaces of nanocomposites. The XPS survey spectra clearly showed the presence of all the dopant materials in the nanocomposites (Figure 4a). The high-resolution XPS cerium compound Ce 3d core-level spectra are depicted in Figure 4b. The core-level spectra were deconvoluted into multiple peaks corresponding to the Ce^4+^ and Ce^3+^ oxidation states. These peaks at 887.7, 891.9, 900.3, 906.1, 910.7, and 918.2 eV represented the Ce^4+^-binding energies. In addition, the other binding energies of Ce^3+^ were at 884.4, 889.4, 903.1, and 908.7 eV. The presence of Ce^3+^ sites on the surface of nanocomposites is crucial for the catalytic performance and regenerative capacity (Ce^3+^ ↔ Ce^4+^) of ceria nanoparticles [31,32,33]. Figure 4c presents the Cu 2p high-resolution spectra of Cu core-level spectra. The Cu 2p_3/2_ and Cu 2p_1/2_ peaks at 935.5 and 954.9 eV, respectively, indicated the formation of the Cu^+^ (Cu_2_O) oxidation state in the nanocomposites. Concurrently, the Cu^2+^ oxidation peaks also formed at 942.1, 946.9, 960.5, and 966.2 eV, indicating the presence of the Cu^2+^ (CuO) oxidation state. The results revealed that the copper was present in two forms (CuO and Cu_2_O) of oxidation states in the nanocomposites. Figure 4d also depicts the high-resolution La 3d core-level spectra. These core-level spectra were deconvoluted into four peaks, all of which originated from the spin-orbital splitting of the La 3d_5/2_ and La 3d_3/2_ states of La^3+^ oxidation. Figure 4e depicts the high-resolution deconvoluted spectra of C1s for Cu/La/CeO_2_/GO nanocomposites, which had four oxygen-related functional groups at 285.8, 287.02, 288.78, and 289.5 eV. These oxygen-related functional groups were attributed to the C–O (hydroxyl), C=O (carbonyl), O–C=O (carboxylic acid), and carbonate bonds, respectively [34,35,36,37,38]. Moreover, the C‒C (aromatic) and π–π interaction bonds were also present at 284.6 and 290.3 eV, respectively.

### 3.4. Photocatalytic Activity of Synthesized Cu/La/CeO_2_/GO Nanocomposites

**Figure 5:**
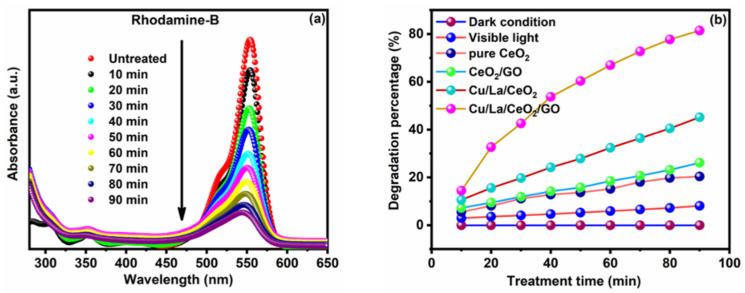
(**a**) Absorption spectrum and degradation percentage of Rhodamine B; (**b**) degradation percentage.

The photocatalytic degradation ability of the hydrothermally synthesized nanocomposites was studied via the degradation of organic pollutants. The photodegradation of harmful organic dyes such as RhB, SY, and CR with nanocomposites and catalyst alone required a long treatment time. Moreover, the degradation process did not occur in the absence of visible light (Figure 5b). The degradation efficiency of the nanocomposites was studied using RhB dye, and the results confirmed that under visible light, the catalyst had higher photocatalytic degradation efficiency. In addition, the degradation of the RhB dye solution by the nanocomposites was in the following order: pure CeO_2_ < CeO_2_/GO < Cu/La/CeO_2_ (Figure S1 in Supplementary Materials). Figure 5a depicts the analysis of the degradation path of RhB at the maximum wavelength of 550 nm [7,8]. The maximum absorption intensity of the RhB decreased with increasing treatment time, indicating that the degradation percentage depended on the treatment time. The degradation percentage of the aqueous solution was calculated by using the following expression [7,8]:

(2) $$\mathrm{Degradation}\;\mathrm{percentage}=\frac{\left(C_{0}-C_{t}\right)}{C_{0}}\times 100$$

where the dye concentrations before and after treatment are denoted as *C*_0_ and *C_t_*.

**Figure 6:**
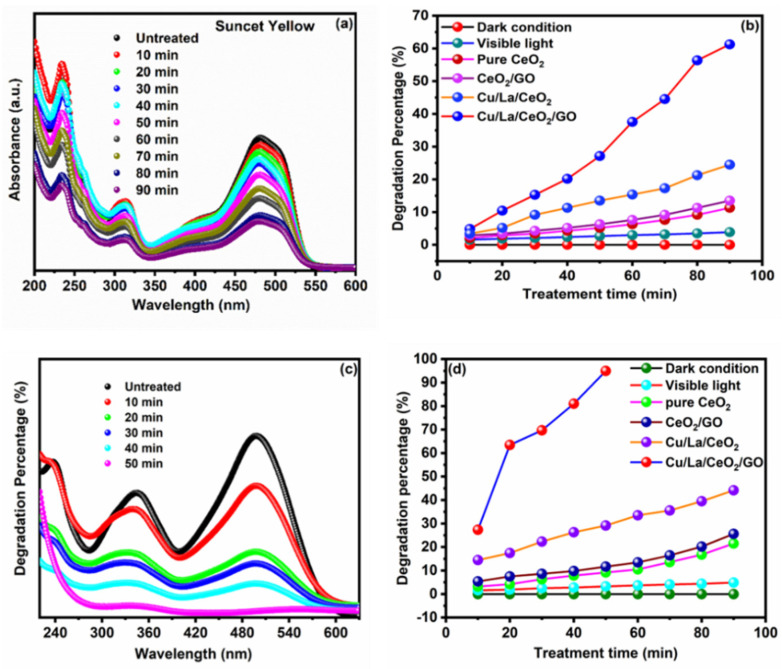
Photodegradation of SY and CR organic dyes using UV–vis spectra. (**a**,**b**) Absorption and degradation percentages of SY. (**c**,**d**) Absorption and degradation percentages of CR.

For a comparative study, the photocatalytic activity of the synthesized nanocomposites was also analyzed for two other toxic dyes, namely, SY and CR. The photocatalytic degradation of SY and CR organic dyes was observed using a UV–visible spectrophotometer at wavelengths of 482 and 500 nm (Figure 6a,c) (Figures S2 and S3), respectively [10,14]. The feature of Cu/La/CeO_2_/GO nanocomposite-assisted photodegradation is the shorter treatment time for the degradation of toxic organic dyes. Thus, it was found that the synthesized nanocomposites were highly efficient for the degradation of harmful organic dyes under visible-light irradiation (Figure 6b,d). Moreover, maximum mineralization was observed in the case of CR dye degradation within 50 min of treatment time. Therefore, because the synthesized nanocomposites tend to form a higher number of electron–hole pairs, higher numbers of hydroxyl and superoxide radicals were generated. These reactive radicals were the major factor in the degradation of the organic pollutants under visible light.

#### 3.4.1. Photocatalytic Degradation Mechanisms 

The catalyst-assisted photocatalytic degradation reaction is an advanced oxidation process organized by a photocatalyst under visible-light irradiation. Pure CeO_2_ is a wide-bandgap material that is active under UV light and allows rapid recombination of photoinduced electron–hole pairs. These are the main reasons for decreases in the photocatalytic ability of the CeO_2_ nanoparticles. The Ce^3+^ ion, which is an excellent photocatalyst, is obtained from the introduction of oxygen vacancies in the Ce^4+^ lattice. Moreover, divalent or trivalent metal ion doping influenced the photocatalytic ability of CeO_2_, resulting in higher degradation activity. Hence, the doping of Cu^2+^/La^3+^ divalent and trivalent ions replaced the Ce^4+^ with Ce^3+^ in the CeO_2_ lattice and created surface defects in the form of oxygen vacancies, while the bandgap of CeO_2_ was also reduced, which increased the light absorption capability in the visible region [19,20,21,22,26]. These oxygen vacancies were largely responsible for the photocatalytic activity of the materials. Moreover, in Cu/La/CeO_2_/GO composites, GO acts as an electron transporter and acceptor because of its two-dimensional planar structure. Thus, the conduction band photogenerated electrons from Cu/La/CeO_2_ transferred to the target pollutant directly with the help of GO. Concurrently, the transferred electrons on GO directly interacted with surface oxygen to produce more superoxide radicals (O_2_^•−^). Additionally, the recombination rate of the photogenerated charge carrier in the CeO_2_ lattice was effectively restricted by the ions of the dopant materials. Therefore, the visible light on the surface of the photocatalyst absorbed more energy, resulting in electrons transferring from the valence band to the conduction band. Photogenerated electron–hole pairs were generated, as were electrons in the conduction band and positive holes in the valence band. The positive holes in the valence band reacted with H_2_O to generate more active hydroxyl radicals (OH^●^), while the excited electrons reacted with adsorbed oxygen to give superoxide radicals (O_2_^●−^). These reactive species were the major reason for the degradation of the targeted organic pollutants. Hence, these reactive species directly interacted with organic molecules, causing oxidation, and excitation occurred. Then the organic molecules transformed into non-hazardous organic molecules, water, and carbon dioxide. The photocatalytic degradation of organic effluents under visible light is expressed in the following equations [27,28,29]:

hν + Cu/Ce/La/GO → CeO (h^+^ + e^−^) + GO (e^−^) (3)

GO(e^−^) + O_2_ → rGO + O_2_^●−^ (4)

e^−^ + O_2_ → O_2_^●−^ (5)

Ce^4+^ + Cu^+^ = Ce^3+^ + Cu^2+^ (6)

Ce^4+^ + La^3+^ → Ce^3+^ (7)

Ce^3+^ + H_2_O_2_ → OH^●^ + OH- + Ce^4+^ (8)

Ce^4+^ + H_2_O_2_ → Ce^3+^ + OH + H_2_O (9)

e^−^ + O_2_ → O_2_^●−^ (10)

O_2_^●−^ + OH^●^ + organic pollutants → H_2_O + CO_2_ (degradation of dyes) (11)

#### 3.4.2. First-Order Kinetic Rate Constant Reaction

**Figure 7:**
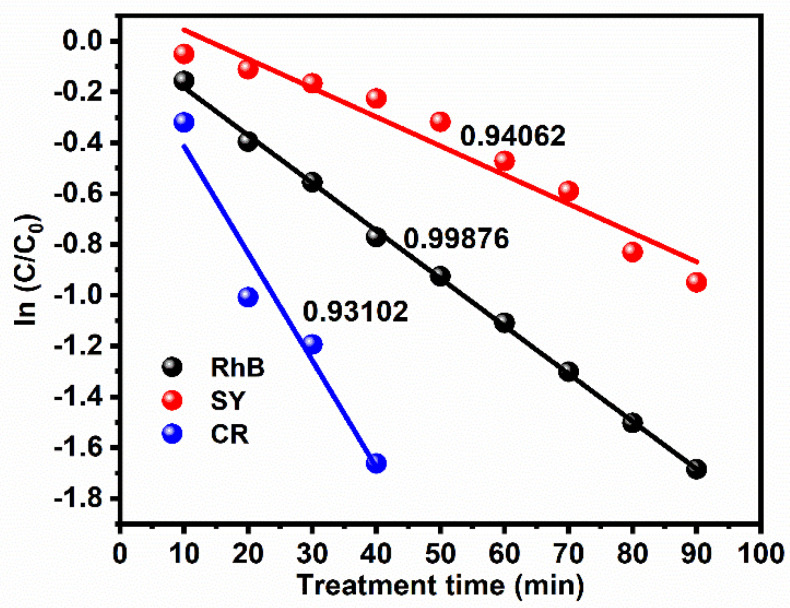
Pseudo-first-order kinetic rate constant for photocatalytic degradation of three different dyes.

The photocatalytic degradation of three different dyes was further evaluated with the help of a pseudo-first-order kinetic rate constant, which can be expressed by the following equation [9]:

ln (C/C_0_) = −kt (12)

where C and C_0_ are the concentrations of dyes before and after the treatment. The rate constant and treatment time are denoted as k and t, respectively. Figure 7 shows the first-order rate constant for the treatment time. The kinetic constants of RhB, SY, and CR were −0.0114, −0.0188, and −0.0421 min, with half-lives of 0.7018, 0.7314, and 0.7445 min^−1^, and the correlation coefficients (R^2^) were 0.9876, 0.94062, and 0.93102, respectively. Therefore, the rate constant mainly depended on the treatment time because the treatment time increased with increases in the rate constant. As a result, the degradation of RhB, SY, and CR was influenced by the generation of higher concentrations of various reactive species [9,10,11,12,13].

#### 3.4.3. Radical-Trapping Experiments

**Figure 8:**
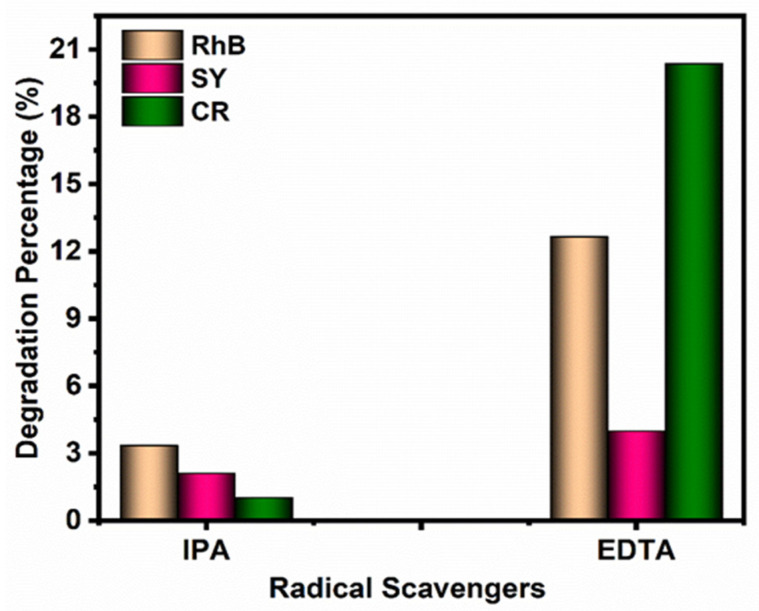
Degradation efficiency of radical scavengers’ experiment.

Photocatalyst-assisted degradation mainly depends on the generation of various reactive species, electrons, and holes. During the degradation reaction, the photocatalyst produces electron–hole pairs under visible-light irradiation, which generates reactive species such as OH^●^, H_2_O_2_, H^+^, O, and O_2_^●−^. These active species play a vital role in the photocatalytic degradation process. Radical scavenger experiments were carried out using the nanocomposites to evaluate the types of reactive species that formed during the photocatalytic degradation process. In this experiment, 2 M of ethylenediaminetetraacetic acid disodium salt (Na_2_EDTA) and isopropanol (IPA) were used as superoxide radicals and hydroxyl radical scavengers, respectively [39,40]. Figure 8 shows the results of the radical scavenger degradation of three different dyes. RhB, SY, and CR dye degradation was higher under visible-light irradiation in the absence of any radical scavengers. However, the degradation efficiency decreased in the presence of Na_2_EDTA and IPA, respectively. The results strongly suggested that OH^●^ was generated as the major active species for the degradation process, though the holes also contributed to the degradation process in the photocatalytic process. 

#### 3.4.4. Nanocomposite Stability Studies 

**Figure 9:**
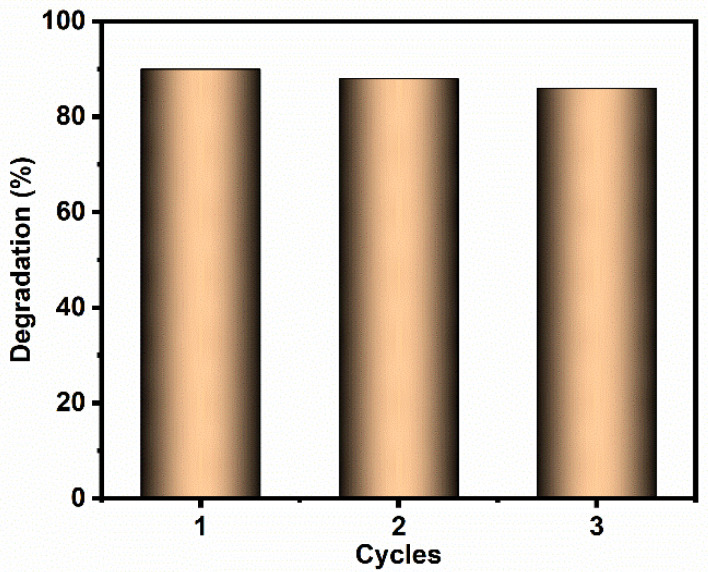
Photocatalytic stability studies of Cu/La/CeO_2_/GO nanocomposites.

To inspect the stability of the synthesized nanocomposites, a recycling experiment was conducted, and the results are depicted in Figure 9. Three successive cycles were conducted for RhB dye degradation under visible-light irradiation using 2 wt% Cu/La/CeO_2_/GO. The synthesized nanocomposite showed excellent efficiency (86%) of RhB dye degradation even in the third cycle, demonstrating the stability of the nanocomposites.

## 4. Conclusions

Cerium oxide (CeO_2_) is a highly efficient photocatalyst, as it absorbs more visible light than TiO_2_ and ZnO. The crystalline structure, surface morphology, and topography of the hydrothermally synthesized nanocomposites were studied. Cerium oxide was engineered by doping with Cu and La metal ions to increase visible-light absorption. Moreover, the efficiencies of the photocatalytic degradation of RhB, SY, and CR organic dyes were 80% (RhB), 60% (SY), and 95% (CR) with treatment times of 90, 90, and 50 min, respectively. The photocatalytic degradation was improved by doping the metal ions, as well as the graphene oxide, due to the higher electron mobility, high light absorption capability, and restrictions of electron–hole pair recombination. In this regard, higher amounts of various reactive radicals were generated, which facilitated the effective degradation of the organic compounds. Accordingly, the hydrothermally synthesized Cu/La/CeO_2_/GO nanocomposites appear to be promising photocatalysts for the degradation of toxic organic dyes in aqueous solution.

## Data Availability

Data are contained within the article.

## Supplementary Materials

The following are available online at www.mdpi.com/article/10.3390/ma14206143/s1, Figure S1: (**a**–**c**) Absorption spectrum of Rhodamine-B dye for various catalysis with re-spect to treatment time, Figure S2: (**a**–**c**) Absorption spectrum of sunset yellow dye for various catalysis with respect to treatment time, Figure S3: (**a**–**c**) Absorption spectrum of cibacron red dye for various catalysis with respect to treatment time.
