# Supplementary material for: Environmental Remediation of Toxic Organic Pollutants Using Visible-Light-Activated Cu/La/CeO2/GO Nanocomposites
Source: Materials (Basel). 2021 Oct 16;14(20):6143. doi: 10.3390/ma14206143 (PMC8537214; doi:10.3390/ma14206143)
Supplement: Supplementary file 1 [file materials-14-06143-s001.zip › materials-1388079-supplementary.pdf]

materials

Supplementary Materials

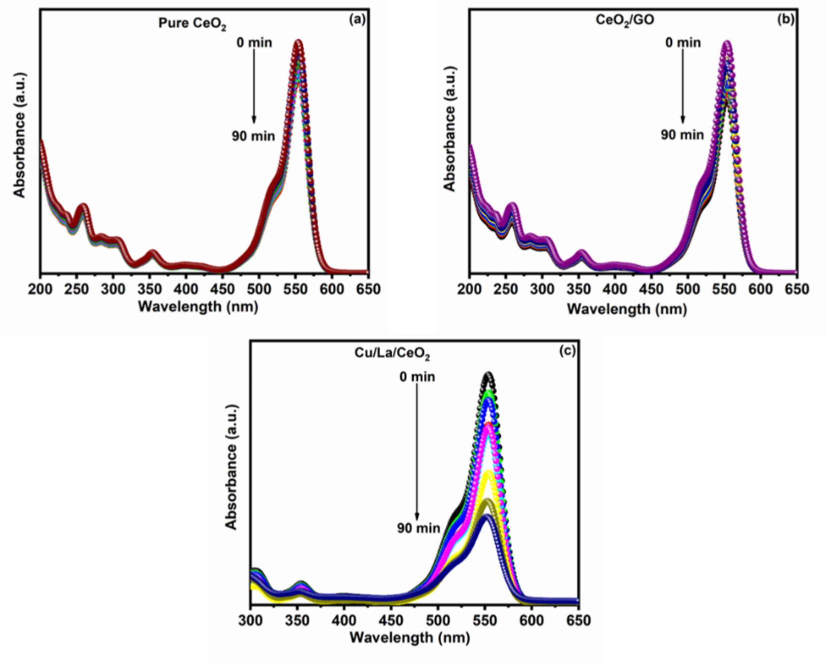

Figure S1. (**a**, **b**, and **c**) Absorption spectrum of Rhodamine-B dye for various catalysis with respect to treatment time.

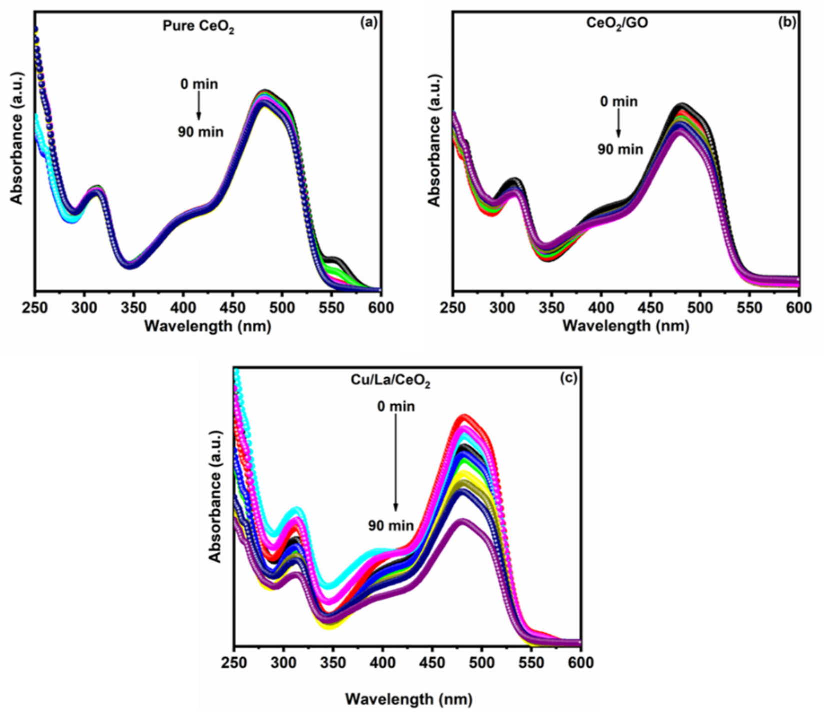

Figure S2. (**a**, **b**, and **c**) Absorption spectrum of sunset yellow dye for various catalysis with respect to treatment time.

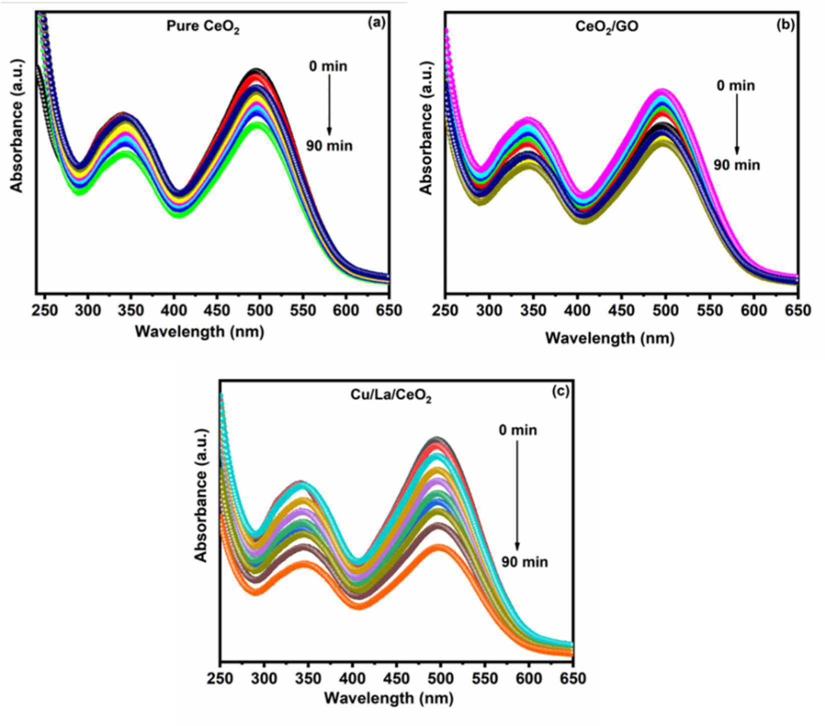

Figure S3. (**a**, **b**, and **c**) Absorption spectrum of cibacron red dye for various catalysis with respect to treatment time. .
